# A Masquerading Hematoma Resulting in the Delayed Diagnosis of a Soft Tissue Sarcoma: A Case Report

**DOI:** 10.7759/cureus.44822

**Published:** 2023-09-07

**Authors:** Matthew E Wells, Jesse Qiao, Katelyn E Decker, Nata Parnes, Rajiv Rajani, Michael Eckhoff

**Affiliations:** 1 Department of Orthopedic Surgery and Rehabilitation, Texas Tech University Health Sciences Center El Paso, El Paso, USA; 2 Department of Pathology, University of California Irvine, Irvine, USA; 3 Department of Orthopaedic Surgery and Rehabilitation, Carthage Area Hospital, Carthage, USA; 4 Orthopaedic Surgery, Claxton-Hepburn Medical Center, Ogdensburg, USA; 5 Department of Orthopedic Surgery and Rehabilitation, Texas Tech University Health Sciences Center El Paso Paul L. Foster School of Medicine, El Paso, USA

**Keywords:** hematoma formation, expanding hematoma, bicep tendon, malignant misdiagnosis, sarcoma soft tissue, rhabdomyosarcoma (rms), soft tissue, misdiagnosis, sarcoma, soft tissue sarcoma

## Abstract

A 27-year-old male with insidious right arm swelling was diagnosed with a hematoma secondary to a partial biceps tear, later identified as a rhabdomyosarcoma. Soft tissue sarcomas (STS) may present with misleading patient histories and nonspecific symptoms, resulting in misdiagnosis and delayed treatment. One of the classic masqueraders of soft tissue sarcomas is hematomas secondary to trauma. Obtaining a prudent history with careful scrutiny of appropriate imaging often helps establish the correct diagnosis. Ultimately, tissue biopsy can resolve any ambiguous cases and prevent delays in diagnosis and treatment.

## Introduction

Soft tissue sarcomas (STS) are mesenchymal-derived malignancies with varying clinical behavior, anatomic location, and histologic/molecular characteristics. These tumors are generally rare, accounting for less than 1% of all adult cancers and 7-10% of pediatric cancers [1]. They often present occultly with nonspecific symptoms, with the most common delay in treatment attributed to initial misdiagnoses.

Previous reports of soft tissue sarcomas being misdiagnosed as hematomas [2-5] and vice versa [6-9] are present in the literature. The reason for misdiagnosis is believed to be due to insufficient information obtained with ultrasound [10] or similarities found on MRI between chronic expanding hematomas (CEH) and STS [11]. These similarities include heterogeneous signals on T1 and T2-weighted imagining, capsule formation, and peripheral enhancement with contrast. It is of the utmost importance to obtain a complete history and physical examination with clinical correlation to imaging findings. It would be unsurprising for patients on anticoagulative medications to present with a chronic hematoma secondary to incidental trauma. However, the same conclusions in a healthy individual not on anticoagulative medications and not having a genetic predisposition affecting coagulation (i.e., hemophilia) would raise concerns about an underlying neoplastic process. Therefore, in ambiguous cases with concern for STS, a tissue biopsy diagnosis should be obtained using sound biopsy principles by an experienced musculoskeletal oncologist or interventional radiologist within a multi-disciplinary care center [12].

## Case presentation

A 27-year-old male presented to the emergency department with right upper extremity swelling that had been waxing and waning. He stated that the swelling began after carrying heavy boxes during a move approximately one month prior. However, he denied any known trauma to his arm. The patient thought that he had sustained a bicep strain or had torn another muscle, but given the lack of ecchymosis, he did not seek medical attention. However, after a month of continued swelling, he presented for further evaluation. His primary concern was that his range of motion was becoming limited and he was having difficulty performing activities of daily living due to the tension in his arm. He denied pain or paresthesia. He worked in construction, was not on any medications, and denied any illicit drug use. On clinical examination, there was no ecchymosis present, and he had limited terminal elbow flexion. The involved brachium was appreciably larger than the contralateral side, but otherwise, there were no significant findings.

**Figure 1:**
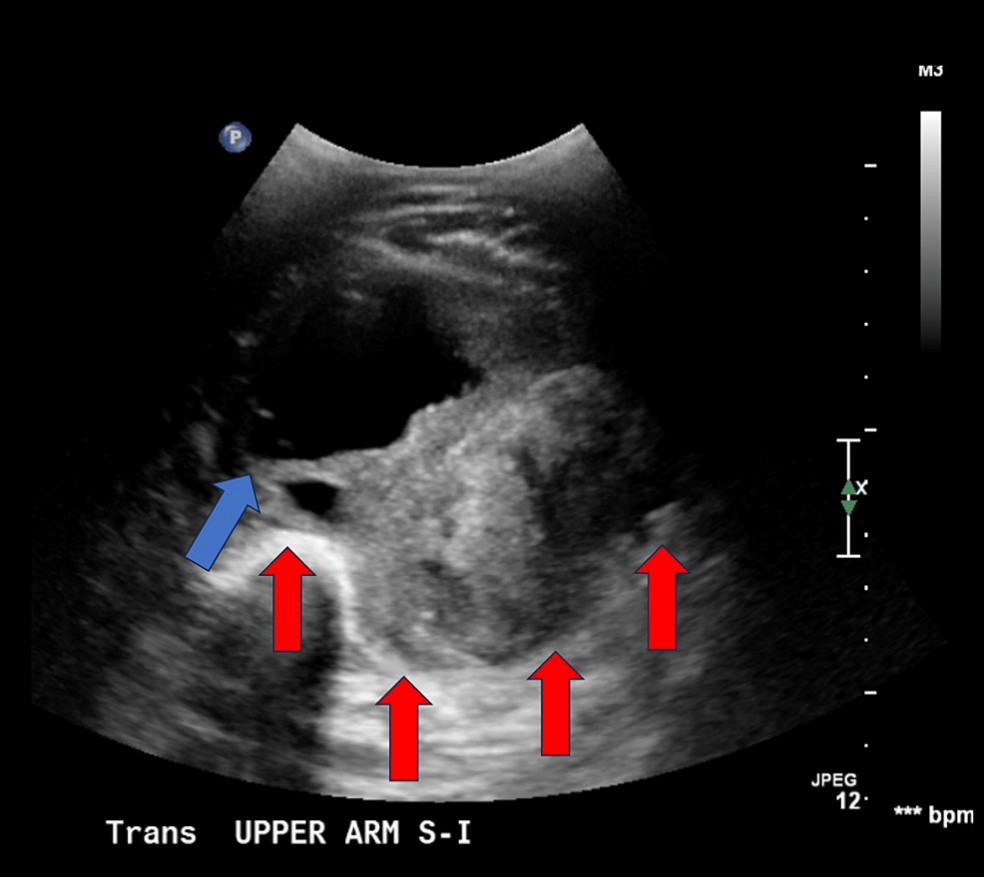
Ultrasound displaying a heterogenous nonvascular mass measuring 13.4 cm× 8.6 cm × 8.1 cm (red arrows indicating borders of the pseudocapsule, blue arrows indicating peritumoral edematous fluid accumulation). The radiologist reported, “negative for deep venous thrombosis with the primary differential consideration of evolving hematoma and myositis.”

**Figure 2:**
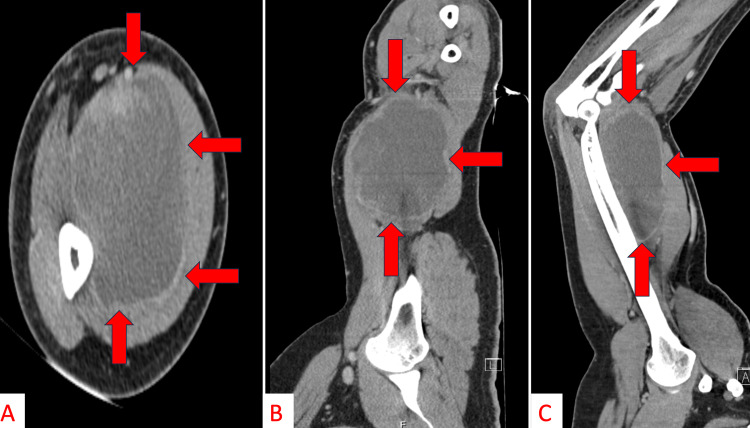
Axial (A), coronal (B), and sagittal (C) CT-scan images with contrast displaying a mildly irregularly marginated/enhancing heterogeneous lesion measuring up to 15 cm overlying the distal anteromedial humerus (red arrows indicate borders of the pseudocapsule). The radiologist reported, “the primary differential considerations included evolving hematoma and Morel Lavallee lesion given the recent history of trauma, cannot exclude an evolving abscess."

He underwent an ultrasound examination (Figure 1) and a computed tomography (CT) scan with iohexol contrast (Figure 2). Based on the CT scan results, he was initially diagnosed with a hematoma versus an abscess after being evaluated by the emergency medicine physician and the general surgery service. Orthopedic surgery was consulted and recommended admission for magnetic resonance imaging (MRI) studies to better evaluate the arm due to concern for possible malignancy.

**Figure 3:**
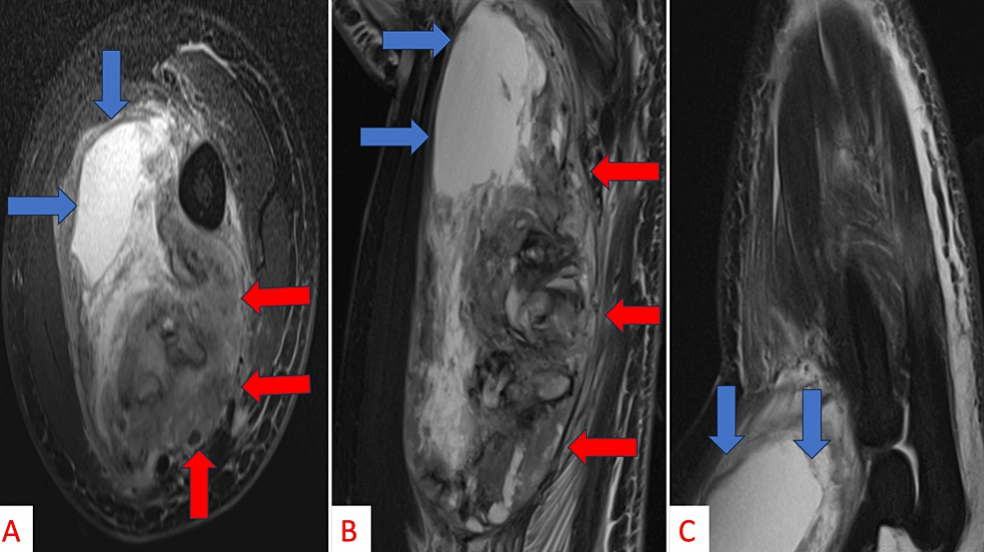
Axial (A), coronal (B), and sagittal (C) MRI imaging displaying a heterogenous mass with irregular enhancing nodular soft tissue and a large cystic/necrotic component consistent with a soft tissue sarcoma (red arrows indicate borders of the pseudocapsule, blue arrows indicate peritumoral edematous fluid accumulation).

He was first admitted by the general surgery service but discharged without an MRI evaluation. Due to the over-filled hospital capacity, the admitting team felt that the MRI could be obtained as an outpatient with routine follow-up in the orthopedic clinic. The following day, he was called back into the ER by the orthopedic surgery service, subsequently readmitted, and underwent an MRI evaluation (Figure 3). The MRI was concerning for soft tissue sarcoma. The patient underwent an appropriate malignancy workup with CT chest/abdomen/pelvis, revealing no appreciable macrometastases, and an ultrasound-guided core-needle biopsy of the mass, which was sent for microbiological culture and tissue evaluation (i.e., cytogenetics).

**Figure 4:**
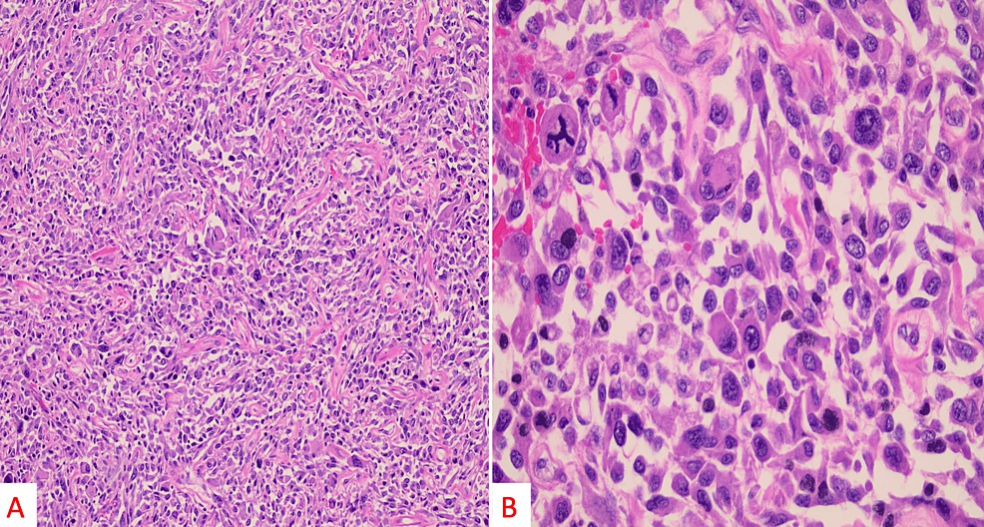
On medium power evaluation (H&E, 100×), the tumor showed hyperchromatic and pleomorphic cells with occasional multinucleated giant cells displaying variable nuclear to cytoplasmic ratios (A). On high power evaluation (H&E, 400× magnification), mitoses are readily identified (including bizarre and atypical/tripolar form; B).

**Figure 5:**
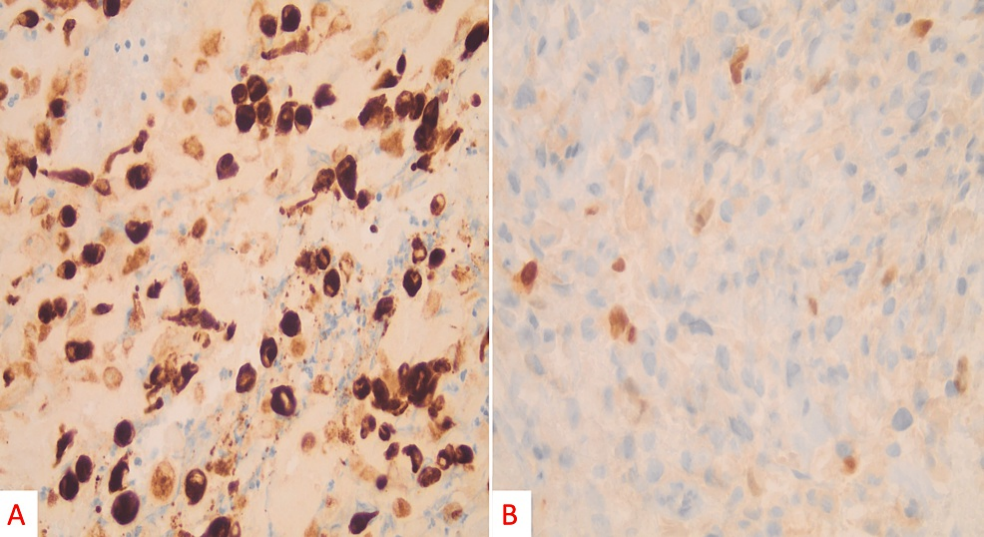
On high power immunostaining (400×, myf-4), the tumor was diffusely positive for desmin (A) and had variable positive expression of myogenin (B).

Histological sections showed an overtly malignant pleomorphic, spindled, epithelioid, and rhabdoid sarcoma growing within an admixture of hyalinized stromal collagen and abundant extravasated blood. On medium-power evaluation, the tumor showed hyperchromatic and pleomorphic cells, with occasional multinucleated giant cells displaying variable nuclear-to-cytoplasmic ratios (Figure 4A). Mitoses are readily identified (including bizarre and atypical/tripolar forms) at high power (Figure 4B). On immunostaining, the tumor was diffusely positive for desmin (Figure 5A) and had variable positive expression of myogenin (Figure 5B). CD31, CD34, myosin, S100, and pankeratin were negative. Smooth muscle actin and caldesmon also showed variable staining. Given the clinical presentation, tumor morphology, and immunoprofile, a diagnosis of a high-grade pleomorphic adult-type rhabdomyosarcoma was made.

**Figure 6:**
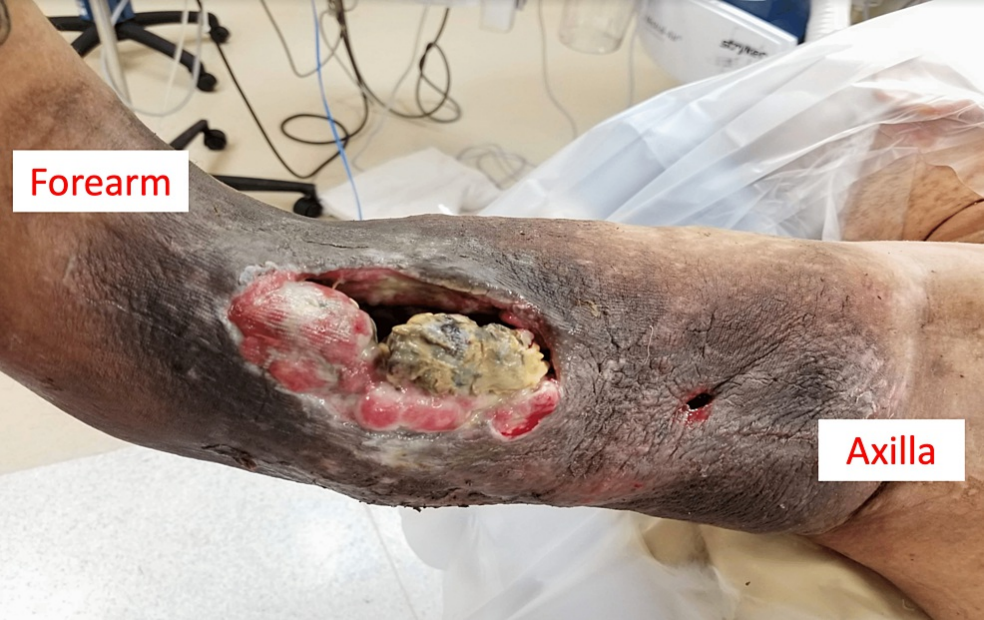
Clinical photographs of the patient’s fungating pleomorphic rhabdomyosarcoma ten weeks into chemotherapy at which point he underwent a glenohumeral disarticulation.

His final diagnosis was stage III pleomorphic (adult-type) rhabdomyosarcoma. The patient underwent five cycles of neoadjuvant chemotherapy over the next two months with doxorubicin, ifosfamide, and sodium 2-mercaptoethane sulfonate (mesna). While on chemotherapy, however, the patient had continued enlargement of the tumor with fungating skin breakdown (Figure 6), secondary to a poor clinical response to the chemotherapy regimen. He ultimately required amputation via glenohumeral disarticulation. There was approximately 20-30% tumor necrosis, with tumor viability estimated at 70%. The final resection tumor margins were confirmed negative by histology. At no time has the patient demonstrated evidence of metastatic disease. Adjuvant chemotherapy treatment was with vincristine, actinomycin D, and cyclophosphamide.

## Discussion

Soft tissue sarcomas can present as insidious, benign masses and are often misdiagnosed as other masquerading conditions [13-15]. In the case of a soft tissue "hematoma" that is not associated with trauma, has no history of ecchymosis, and is increasing in size or symptoms, concerns arise about the possibility of soft tissue sarcomas [16]. Obtaining a thorough history and physical examination in conjunction with advanced imaging is the most prudent initial approach. Should any ambiguity remain, the patient should be referred urgently to a trained musculoskeletal oncologist at a specialized treatment hospital for further evaluation [17].

The first step in evaluation is obtaining a thorough patient history and physical examination. The mass history of when it was identified, if it is growing, and at what rate may provide an early clinical assumption. A small mass that has been present for years is likely benign, as opposed to a rapidly enlarging mass. The anatomic location (body part, deep vs. superficial to fascia), mass size, and palpable consistency (soft vs. firm) should be noted and correlated to the patient’s history. Generally, larger masses (>3 cm) that are deep in the fascia and/or enlarging in size warrant urgent advanced imaging evaluation. There is a general lack of experience in the evaluation of soft tissue masses among generalist providers with ambiguity in presentation. Fortunately, the Musculoskeletal Tumor Society (MSTS) recognizes these limitations and provides clinical practice guidelines on its website for public use and clinical decision assistance. They recommend that all soft tissue masses that are appreciably enlarging or are baseline larger than 5 cm have a significant likelihood of being malignant and should be promptly referred to a sarcoma specialist [18]. They further state that obtaining an MRI of the entire tumor with and without contrast prior to referral is appropriate. Therefore, smaller masses that are not rapidly growing can and should undergo further evaluation by the initial treatment provider.

Advanced imaging can often help differentiate between hematoma and STS; however, results may be inconclusive [10]. MRI and PET scans may fail to differentiate between chronic hematoma and STS [8,11,19]. However, more recent studies demonstrate improved diagnostic potential with MRI through the use of diffuse weighted imaging, where differences in apparent diffusion coefficient (ADC) are observed [11]. At this point, the decision must be made to refer to a sarcoma specialist for a potential biopsy or interval follow-up to ensure no further changes occur with continued observation.

It is imperative that a biopsy be performed by a trained sarcoma specialist. There are significant potential morbidities [17,20], and even worse mortality associated with inappropriate biopsy approach/technique or unplanned excisions of soft tissue sarcomas. In some patients, multiple biopsies or an open biopsy may be required, as tumors associated with hematomas may only have malignant cells in the capsule or peripheral tissues [4].

The confirmation of diagnoses by pathology may require a combination of modalities to examine the tissue. After obtaining an adequate biopsy specimen, evaluation of tumor morphology will guide the selection of subsequent ancillary testing [21]. Ancillary testing in STS pathology typically includes immunohistochemical stains to categorize the lineage of the malignant cells or the use of molecular methods (cytogenetics, fluorescent-in-situ hybridization, or sequencing) to detect recurrent genetic abnormalities with the tumor, if present. The diagnosis of STS requires careful clinical and imaging correlation, in particular with patient age. While this patient presents with an adult-type rhabdomyosarcoma that does not have recurrent genetic alterations, pediatric rhabdomyosarcoma subtypes may present with recurrent chromosomal translocations or known karyotype alterations [22].

Case reports and series have demonstrated similar presentations to this article. Kontogeorgakos et al. performed a retrospective review of 15 patients who were initially diagnosed with hematomas and later diagnosed with STS [5]. Most of their patients (11 of 15) were later diagnosed with an undifferentiated pleomorphic sarcoma (UPS, previously classified as malignant fibrous histiocytoma), with the others having a synovial sarcoma (2), angiosarcoma (1), and rhabdomyosarcoma (1). To the best of our knowledge, Ward et al. [2] published the largest case series (31 patients), with 21 MRI studies initially reporting no concern for any malignant processes. The most common tumor identified in the study was also UPS in 11 of 31 patients, with the thigh being the most common location.

## Conclusions

Differentiating a hematoma from a soft tissue sarcoma is appreciably difficult, with biopsy remaining the gold standard diagnostic modality. Advanced imaging using MRI with and without contrast should be obtained. Watchful waiting is appropriate in smaller (<5 cm) masses that do not continue to grow so long as the patient is adherent to interval follow-up appointments. Any growing soft tissue masses or those initially >5 cm in size should be promptly referred to a sarcoma specialist for diagnostic biopsy and definitive treatment.
